# The Annual Report for 1885 of the Surgeon-General of the Navy

**Published:** 1885-12

**Authors:** 


The Annual Report of the Surgeon-General of the United States Navy, is comprised in a pamphlet of nine pages, which, being printed at the Government Printing Office instead of by the Bureau itself, compares unfavorably in typographical appearance with the Army Report. In the force afloat for the year, represented by the figures 9,959, there were 78,659 entries of sick on the rolls, the traditions of the navy being such that a man who is ill for six days counts as six men in the same condition for one day. The order of diseases represented in the list is, first, violent diseases and deaths; next, constitutional diseases; and then, diseases of the genito-urinary system; of the integumentary system; of the digestive system; zymotic diseases; respiratory diseases, etc. The difference between the health of the army and of the navy with respect to the class of disorders affecting the respiratory organs is most marked, and is highly suggestive of the salubrity of the sea and the modifications impressed by it upon the climate.

Special attention should be directed to the museum of hygiene under the charge of Medical-director Browne. A large number of contributions have been made to it during the year, such as articles illustrating drainage, plumbing, water-supply, ventilation, household-health, disposing of the dead, etc., and books, instruments, etc.

On the outside of the museum is being constructed a complete system of iron and lead pipes, with fixtures, running from the ground to the roof, with an observing station at each of the three stories, for an exhaustive series of experiments covering all the topics in dispute relating to trap-siphonage, and the utility of the mechanism of water-closets, traps, water-basins, baths, sinks, etc. The results of this novel enterprise on the part of the Bureau will be watched with interest, as the experiments will be conducted in the cause of science alone, uninfluenced by any of the commercial interests which, it is feared, have too often given coloring to results supposed to have been obtained in experiments, presumably conducted in the interest of sanitary science. The work in this museum has been conducted without ostentation, and but few are aware how much this collection of models and plans and sanitary devices will yet influence sanitation at sea and on land. A liberal appropriation by congress for the further development of the work undertaken in the formation of this valuable museum, will enable those directing it to carry out their cherished plan of making it useful in the direction of prevention of sickness, rather than of its remedy, which is now engaging the attention of sanitarians, who recognize in it the highest calling of the physician.

